# Association of relative hand grip strength with myocardial infarction and angina pectoris in the Korean population: a large-scale cross-sectional study

**DOI:** 10.1186/s12889-024-18409-w

**Published:** 2024-04-02

**Authors:** Jeong Hee Chi, Bum Ju Lee

**Affiliations:** 1 Department of Computer Science and Engineering, Konkuk University, Seoul, Republic of Korea; 2 Digital Health Research Division, Korea Institute of Oriental Medicine, 1672 Yuseong-daero, Yuseong-gu, 34054 Daejeon, Republic of Korea

**Keywords:** Acute coronary syndrome, Cardiovascular disease, Myocardial infarction, Angina pectoris, Risk factor, Anthropometry

## Abstract

**Background:**

Low hand grip strength (HGS) is associated with the risk of cardiovascular diseases, but the association between HGS and myocardial infarction/angina pectoris (MIAP) is unclear. Furthermore, there have been no studies examining the associations of MIAP with anthropometric indices, absolute HGS indices, and relative HGS indices calculated by dividing absolute HGS values by body mass index (BMI), waist circumference (WC), waist-to-height ratio (WHtR), or weight values. Therefore, the objective of this study was to examine the associations of MIAP with absolute and relative HGS combined with several anthropometric indices.

**Methods:**

In this large-scale cross-sectional study, a total of 12,963 subjects from the National Health and Nutrition Examination Survey were included. Odds ratios and 95% confidence intervals for the associations of MIAP with anthropometric indices, absolute HGS indices, and relative HGS indices were computed from binary logistic regression models. We built 3 models: a crude model, a model that was adjusted for age (Model 1), and a model that was adjusted for other relevant covariates (Model 2).

**Results:**

For men, the average age was 61.55 ± 0.16 years in the MIAP group and 66.49 ± 0.61 years in the non-MIAP group. For women, the average age was 61.99 ± 0.14 years in the MIAP group and 70.48 ± 0.61 years in the non-MIAP group. For both sexes, the MIAP group had lower diastolic blood pressure, shorter stature, greater WC, and a greater WHtR than did the non-MIAP group, and women tended to have greater systolic blood pressure, weight, and BMI than in men. HGS was strongly associated with the risk of MIAP in the Korean population. In men, relative HGS indices combined with WC and the WHtR had greater associations with MIAP than did the anthropometric indices and absolute HGS indices. However, in women, anthropometric indices, including weight, BMI, WC, and WHtR, were more strongly associated with MIAP than were absolute and relative HGS indices, unlike in men. When comparing absolute and relative HGS indices in women, relative HGS indices combined with BMI and weight was more strongly related to MIAP than was absolute HGS indices.

**Conclusions:**

MIAP might be better identified by relative HGS than absolute HGS in both sexes. The overall magnitudes of the associations of MIAP with absolute and relative HGS are greater in men than in women.

## Introduction

Myocardial infarction (MI) is an important cause of mortality and disability worldwide [1–3]. MI is defined as myocardial cell necrosis, vascular occlusion, or thrombosis according to the status of long-term ischemia and the complexities of the cellular functions of inflammation and scar formation [1–3]. MI is the first manifestation of coronary artery disease (CAD) and is one of several critical manifestations (angina pectoris, heart failure, and unexpected death) of coronary heart disease [2, 3]. Customarily, the concept of acute coronary syndrome (ACS) includes patients with unstable angina pectoris, ST-segment elevation MI, or non-ST-elevation MI according to clinical criteria [2, 3].

Recently, low hand grip strength (HGS) was strongly associated with the risk of several diseases, such as MI/angina pectoris (MIAP) [4–15], stroke [5–7, 9], heart disease [12], nonalcoholic fatty liver disease [16], myocardial ischemia [17], diabetes [13, 18, 19], metabolic syndrome [20], depression [21, 22] and pulmonary dysfunction [23], cardiovascular mortality and noncardiovascular mortality [4–7, 9, 10], cardiovascular health biomarkers [14, 15], and quality of life [24]. However, the associations between HGS and MIAP or cardiovascular risks are unclear. Many studies have reported that low absolute HGS is a strong predictor of MIAP in many countries [4–12], whereas some studies have argued that HGS is not associated with cardiovascular risk factors or cardiovascular mortality [25–27]. On the other hand, more recently, several studies examined the association between relative HGS (HGS divided by weight, height, or body fat mass) and several diseases, such as cancer [28], type 2 diabetes [13], cardiometabolic disease [14, 15], and metabolic syndrome [20]. Although many studies have examined the association between the absolute HGS index and MIAP, to our knowledge, there have been no studies examining the association between MIAP and both absolute and relative HGS indices. Therefore, the objectives of this study were to examine the associations of MIAP with anthropometric indices, absolute HGS indices, and relative HGS indices combined with several anthropometric indices and to identify the indices most strongly associated with this disease.

The originality and significance of this study are that it is the first to compare the association between MIAP and absolute and relative HGS combined with various anthropometric indices and to demonstrate that relative HGS is more strongly associated with MIAP than is absolute HGS in both men and women in a large-scale Korean population.

## Methods

### Study design and population

**Figure 1:**
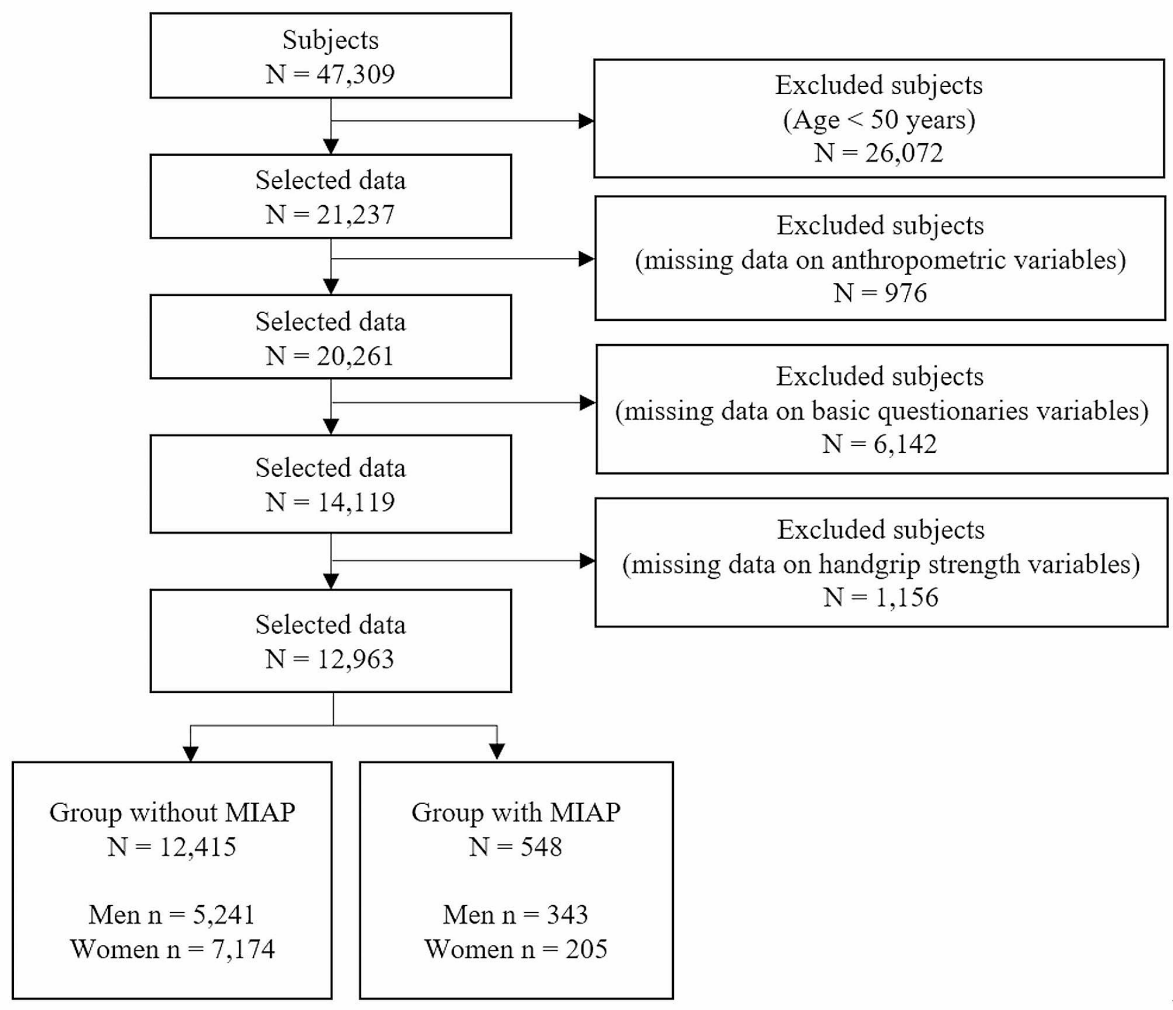
Sample selection procedure used in this study

In this large-scale cross-sectional study, we utilized the Korea National Health and Nutrition Examination Survey (KNHANES) dataset provided by the Korea Disease Control and Prevention Agency (KDCA). The KNHANES is a nationwide health and nutrition survey conducted annually to produce statistics with national representativeness and reliability on the health status, health behavior, and food and nutritional intake of the population. The survey collects data through health interview surveys, health examination surveys, and nutrition surveys [29–31]. In this study, we used recent HGS and MIAP data from 2014 to 2019. The data from 2020 were not included in this study, as the measurement of HGS was suspended due to the spread of COVID-19. From 2014 to 2019, a total of 47,309 participants (men = 21,566, women = 25,743) participated in the health interview survey and examination conducted by mobile examination vehicles. The target participants of this study were adults aged 50 years or older in South Korea. Initially, participants were selected based on age, and participants with missing data on anthropometric variables, basic questionnaire variables, and HGS variables were excluded. Further details regarding the participant selection process are visually presented in Fig. 1. Ultimately, a total of 12,963 participants who were not missing data for the study variables were included.

### Ethics approval

The KNHANES was approved by the Research Ethics Committee of the KDCA (IRB: 2013-07CON-03–4 C, 2013-12EXP-03–5 C, 2018-01-03-P-A, 2018-01-03-C-A). This study, which was based on the KNHANES dataset, was also ethically approved by the Institutional Review Board of the Korea Institute of Oriental Medicine (IRB No. I-2209/009 − 001). This study was conducted in accordance with the Helsinki Declaration, and all methods followed relevant guidelines and regulations [29–31].

### Definitions of MIAP

The primary outcome of this study was MIAP. We defined MIAP patients as a single MIAP group according to previous methods in various cross-sectional and follow-up studies [32–36]. The presence of MIAP was determined by two questions included in the health interview survey: “Have you been diagnosed with MI by a doctor?” and “Have you been diagnosed with angina by a doctor?“. Participants who answered “yes” to either question were included in the MIAP group, and those who answered “no” to both questions were included in the non-MIAP group. To overcome respondent recall bias in the diagnosis of MIAP, the health interview survey was carried out through a face-to-face interview with experts and well-trained staff according to the established guidelines [29–31]. The status of other diseases, such as hypertension, diabetes, hypercholesterolemia, and hypertriglyceridemia, was determined using blood tests and health interview survey data according to the KDCA guidelines [31]. Hypertension was defined as a systolic blood pressure (SBP) of 140 mmHg or higher, a diastolic blood pressure (DBP) of 90 mmHg or higher, or the current use of antihypertensive medication [37, 38]. Diabetes status was defined as a fasting plasma glucose level of 126 mg/dl or higher, a glycated hemoglobin level of 6.5% or higher, the current use of antidiabetic medication, the use of insulin injection therapy, or a diagnosis of diabetes by a doctor [38, 39]. Hypercholesterolemia was defined as a fasting total cholesterol level of 240 mg/dl or higher or the current use of cholesterol-lowering medication [40, 41]. Hypertriglyceridemia was defined as a fasting triglyceride level of 200 mg/dl or higher [41, 42].

### Covariates

We included demographic and health behavior-related variables such as residential area, education level, occupation type, household income, stress, alcohol consumption, smoking status, family history of ischemic heart disease (IHD), resistance exercise, and walking exercise as covariates. The demographic data were collected through an interview method, while the health behavior-related data were collected through a self-administered questionnaire. Residential areas were classified into two categories based on the subjects’ current residential location: urban and rural. Education level was categorized into four categories, ranging from an elementary school or lower, middle school, high school, and university or higher. Occupation type was divided into seven categories, ranging from unemployed to white-collar workers, office workers, service workers, farmers or fishers, blue-collar workers, elementary occupations, and unemployed. Household income was divided into four levels as equivalent income quartiles based on average monthly income, and stress was classified into four groups (extremely, very, slightly, and rarely) according to the degree of perceived stress. Alcohol consumption was categorized into seven levels (never drinker, former drinker 1 year prior, < 1 drink per month, 1 drink per month, 2∼4 drinks per month, 2∼3 drinks per week, and > = 4 drinks per week) based on the frequency of alcohol consumption in the past year, and smoking status was classified into three groups (current smoker, former smoker, and never smoker) according to current and past smoking status [29–31]. Information on family history of IHD was obtained through the question “Have your father, mother, or siblings ever been diagnosed with ischemic heart disease by a doctor?” Resistance exercise was classified into four categories (never, 1∼2 days per week, 3∼4 days per week, and > = 5 days per week) based on the question “How many days did you perform strength training, such as push-ups, sit-ups, dumbbell training, kettlebell training, or barbell training, in the past week?“, and walking exercise was expressed in minutes per week.

### Measurement

In this study, we analyzed the associations between MIAP and variables related to HGS and anthropometric indices. HGS and anthropometric data were collected by trained examiners who received specific education and training in strict standardization and quality control measures. HGS was measured after excluding subjects who had a history of hand/wrist surgery or who had experienced functional limitations or discomfort in the last three months, which could make it difficult to measure HGS. A digital HGS dynamometer (T.K. K 5401, Japan) was used to measure HGS; the subjects stood upright, their feet were shoulder-width apart, and they faced forward, with their shoulders naturally lowered so that their elbows and wrists did not touch their torso or bend. The measurement began with the dominant hand, taking a 1-minute break between hands and repeating the measurement three times per hand. Absolute HGS was measured as the maximum HGS of the dominant hand (MaxGS-DH), the maximum HGS of the nondominant hand (MaxGS-nonDH), the maximum HGS of both hands (MaxGS-BHs) and the average HGS of both hands (meanGS-BHs). Relative HGS was calculated by dividing the absolute HGS values by body mass index (BMI), waist circumference (WC), waist-to-height ratio (WHtR), and weight values [43, 44]. For the anthropometric variables, height and weight were measured using an automatic measuring device (JENIX DS-102, Dong Sahn Jenix Co., Seoul, Korea) in units of 0.1 cm and 0.1 kg, respectively. BMI was computed by dividing weight (kg) by height squared (m^2^). WC was measured using a tape measure (Seca 200, Hamburg, Germany) in units of 0.1 cm, and the WHtR was obtained by dividing WC by height. SBP and DBP were measured thrice using a standard mercury sphygmomanometer (Baumanometer Wall Unit 33(0850)/USA) and then averaged using the second and third measurements.

### Statistical analysis

To obtain a representative sample of the entire South Korean population, the KNHANES utilized data from the Population and Housing Census and Joint Housing Price Disclosure as the basis for sample extraction. By applying a two-stage stratified cluster sampling method with survey districts and households as the first and second-stage sampling units, respectively, a representative sample was selected for the KNHANES. Additionally, through household verification surveys, the status of all residences and households in the selected areas was assessed, and households were chosen to participate in health surveys, examinations, and nutritional assessments. In the KNHANES, weights are calculated and provided using information collected from household verification surveys. The basic weights consist of health survey and examination weights, as well as nutritional survey weights. Within the basic weights, separate weights are provided based on the age of the target survey participants, differences in the survey participants, and differences in the survey period. Moreover, considering the simultaneous analysis of multiple variables, the KNHANES also provides association analysis weights that encompass various survey sections, domains, and items. Detailed explanations related to the weights can be found in [31]. In this study, we followed the KDCA guidelines [29–31] and applied health survey and examination weights to conduct complex sample analyses.

All analyses were performed using IBM SPSS Statistics 21 and considering a statistical significance level of 0.05. The characteristics of the subjects were described by dividing men and women into groups with and without MIAP. Categorical variables are expressed as percentages and standard errors, while continuous variables are expressed as the means and standard errors. Sex differences in the analyzed variables were examined using t tests based on general linear models for continuous variables and Rao–Scott chi-square tests for categorical variables.

## Results

### Participant demographic characteristics

The characteristics of the study participants are shown in Table 1. The associations of MIAP with anthropometric indices, absolute HGS indices, and relative HGS indices were analyzed by calculating odds ratios (ORs) and 95% confidence intervals (CIs) through a binary logistic regression model after data standardization. The final sample consisted of 12,963 South Korean adults older than 50 years, including 5,584 (43.08%) men and 7,379 (56.92%) women. The prevalence of MIAP among South Korean adults over 50 years of age was 4.23%, with a higher incidence in men (6.14%) than in women (2.78%). All variables used in the analysis, except for IHD family history, SBP, and BMI, exhibited statistically significant differences between men and women. For the demographic variables, including age, residential area, marital status, education level, occupation type, and household income, significant differences were observed between the non-MIAP group and the MIAP group in all variables, except for residential area and marital status in men and residential area in women. The MIAP group tended to be older, have a lower education level, have a higher unemployment rate, and have a lower household income than the non-MIAP group for both sexes. Among the variables related to health behaviors, resistance exercise significantly differed between the MIAP and non-MIAP groups for men (*p* = 0.004) but not for women (*p* = 0.582). The MIAP group tended to perform less resistance exercise than the non-MIAP group. There were significant differences in stress (*p* = 0.044) and alcohol consumption (*p* = 0.001) between the two groups for women but not for men. The MIAP group tended to experience more stress (i.e., “very” and “rarely”) and have lower alcohol consumption than did the non-MIAP group. For both men and women, there were significant differences between the MIAP and non-MIAP groups in terms of disease-related variables such as IHD family history, hypertension, diabetes, and hypercholesterolemia, but not hypertriglyceridemia. A greater proportion of the MIAP group had an IHD family history and hypertension, diabetes, or hypercholesterolemia than did the non-MIAP group.

**Table 1:** Demographic characteristics of the subjects in this study

Variables	Men			Women		
	Non-MIAP	MIAP	*p* value	Non-MIAP	MIAP	*p* value
Numbers	5,241	343		7,174	205	
Age (years) ^*^	61.55 ± 0.16	66.49 ± 0.61	< 0.001	61.99 ± 0.14	70.48 ± 0.61	< 0.001
Residential area^***^			0.973			0.084
Urban	80.10 (1.40)	80.22 (2.73)		81.80 (1.20)	76.50 (3.50)	
Rural	19.90 (1.40)	19.78 (2.73)		18.20 (1.20)	23.50 (3.50)	
Marital status^***^			0.478			< 0.001
Married	91.00 (0.50)	92.20 (1.50)		71.50 (0.70)	55.50 (4.10)	
Single (widowed, divorced, etc.)	9.00 (0.50)	7.80 (1.50)		28.50 (0.70)	44.50 (4.10)	
Education level^***^			0.004			< 0.001
<= Elementary school	20.62 (0.72)	26.80 (2.70)		37.81 (0.79)	63.50 (4.20)	
Middle school	15.51 (0.63)	19.90 (2.60)		15.88 (0.53)	16.50 (3.30)	
High school	31.91 (0.83)	30.70 (3.00)		30.77 (0.71)	15.20 (3.10)	
>= University	31.96 (0.99)	22.60 (2.70)		15.53 (0.63)	4.70 (1.70)	
Occupation^***^			< 0.001			< 0.001
White-collar worker	12.63 (0.63)	5.70 (1.30)		4.80 (0.30)	0.70 (0.50)	
Office worker	9.48 (0.57)	5.50 (1.60)		4.00 (0.30)	0.90 (0.70)	
Service worker	8.62 (0.50)	10.10 (2.80)		16.50 (0.60)	6.10 (1.70)	
Farmer or fisher	8.16 (0.64)	6.50 (1.70)		4.10 (0.40)	3.30 (1.30)	
Blue-collar worker	22.91 (0.77)	15.70 (2.70)		2.70 (0.20)	0.90 (0.60)	
Elementary occupations	8.99 (0.46)	10.40 (2.00)		13.40 (0.50)	12.40 (2.70)	
Unemployed (housewife, etc.)	29.21 (0.78)	46.10 (3.10)		54.40 (0.70)	75.60 (3.50)	
Household income^***^			0.003			< 0.001
Low	18.26 (0.65)	25.20 (2.50)		26.21 (0.70)	47.10 (4.30)	
Middle-low	25.26 (0.74)	29.90 (3.00)		24.82 (0.63)	24.60 (3.40)	
Middle-high	25.72 (0.78)	24.00 (2.90)		23.25 (0.64)	16.50 (2.90)	
High	30.75 (0.93)	20.80 (3.00)		25.73 (0.76)	11.90 (2.60)	
Stress^***^			0.906			0.044
Extremely	2.93 (0.27)	2.75 (1.14)		4.40 (0.30)	4.88 (1.54)	
Very	14.99 (0.61)	16.24 (2.42)		18.40 (0.60)	22.19 (3.53)	
Slightly	59.23 (0.79)	57.03 (3.11)		56.70 (0.70)	45.47 (4.16)	
Rarely	22.86 (0.63)	23.98 (2.62)		20.50 (0.60)	27.45 (3.45)	
Alcohol consumption^***^			0.140			0.001
Never drinker	5.70 (0.40)	6.70 (1.50)		23.50 (0.60)	33.73 (4.01)	
Former drinker 1 year prior	15.40 (0.60)	19.60 (2.30)		22.00 (0.60)	24.32 (3.17)	
< 1 per month	9.50 (0.50)	13.90 (2.60)		23.90 (0.60)	28.34 (3.67)	
1 per month	8.40 (0.50)	7.10 (1.50)		9.80 (0.40)	3.84 (1.31)	
2∼4 per month	23.20 (0.70)	19.90 (2.50)		13.10 (0.50)	5.39 (1.91)	
2∼3 per week	22.60 (0.70)	19.30 (2.70)		5.50 (0.30)	3.35 (1.62)	
>=4 per week	15.20 (0.60)	13.50 (2.30)		2.10 (0.20)	1.02 (0.63)	
Smoking status^***^			0.084			0.746
Current smoker	28.70 (0.80)	25.60 (2.94)		3.20 (0.30)	3.80 (1.40)	
Former smoker	52.10 (0.80)	59.45 (3.46)		3.20 (0.20)	4.10 (1.50)	
Never smoker	19.20 (0.70)	14.94 (2.32)		93.60 (0.40)	92.10 (2.00)	
Resistance exercise^***^			0.004			0.582
Never	66.19 (0.79)	73.40 (2.80)		84.45 (0.52)	87.80 (2.80)	
1∼2 days per week	8.16 (0.46)	10.20 (2.10)		5.26 (0.31)	3.00 (1.60)	
3∼4 days per week	10.61 (0.51)	4.70 (1.20)		5.00 (0.30)	5.00 (2.00)	
>= 5 days per week	15.04 (0.57)	11.70 (1.90)		5.29 (0.30)	4.20 (1.50)	
Walking exercise (min) ^***^	278.7 ± 6.94	243.1 ± 19.46	0.087	248.6 ± 5.11	259.3 ± 34.71	0.760
Menopause						< 0.001
No	-	-		11.10 (0.50)	0.00 (0.00)	
Yes	-	-		88.90 (0.50)	100 (0.00)	
Family history of IHD			0.019			< 0.001
No	93.60 (0.40)	89.20 (2.10)		93.10 (0.40)	82.80 (3.30)	
Yes	6.40 (0.40)	10.80 (2.10)		6.90 (0.40)	17.20 (3.30)	
Hypertension^**^			0.011			< 0.001
No	53.73 (0.81)	45.00 (3.28)		57.00 (0.70)	27.40 (3.70)	
Yes	46.27 (0.81)	55.00 (3.28)		43.00 (0.70)	72.60 (3.70)	
Diabetes^***^			< 0.001			< 0.001
No	80.40 (0.60)	69.30 (3.10)		84.40 (0.50)	67.50 (3.80)	
Yes	19.60 (0.60)	30.70 (3.10)		15.60 (0.50)	32.50 (3.80)	
Hypercholesterolemia^***^			< 0.001			< 0.001
No	77.71 (0.67)	64.50 (3.10)		63.80 (0.70)	42.60 (4.20)	
Yes	22.29 (0.67)	35.50 (3.10)		36.20 (0.70)	57.40 (4.20)	
Hypertriglyceridemia^***^			0.067			0.283
No	78.70 (0.70)	84.00 (2.60)		87.30 (0.50)	83.90 (3.30)	
Yes	21.30 (0.70)	16.00 (2.60)		12.70 (0.50)	16.10 (3.30)	
Blood pressure						
SBP (mmHg)	123.8 ± 0.27	123.9 ± 0.99	0.873	123.3 ± 0.26	132.5 ± 1.31	< 0.001
DBP (mmHg) ^***^	77.82 ± 0.17	73.04 ± 0.68	< 0.001	75.27 ± 0.14	73.60 ± 0.72	0.023
Anthropometrics						
Height (cm) ^***^	168.2 ± 0.11	167.18 ± 0.42	0.012	155.2 ± 0.08	152.9 ± 0.43	< 0.001
Weight (kg) ^***^	68.70 ± 0.17	68.43 ± 0.66	0.693	58.02 ± 0.12	59.58 ± 0.57	0.008
Body mass index (kg/m^2^)	24.25 ± 0.05	24.44 ± 0.17	0.273	24.09 ± 0.05	25.51 ± 0.23	< 0.001
Waist circumference (cm) ^***^	86.99 ± 0.14	88.81 ± 0.52	0.001	81.91 ± 0.15	86.80 ± 0.63	< 0.001
Waist-to-height ratio^***^	0.52 ± 0.00	0.53 ± 0.00	< 0.001	0.53 ± 0.00	0.57 ± 0.00	< 0.001
Dominant hand^*^			0.434			0.099
Right	87.62 (0.61)	90.34 (1.91)		89.60 (0.40)	93.80 (1.80)	
Left	5.17 (0.37)	4.24 (1.44)		4.20 (0.30)	1.60 (1.00)	
Both	7.21 (0.47)	5.42 (1.32)		6.20 (0.40)	4.60 (1.60)	
Absolute HGS						
MaxGS-DH (kg) ^***^	38.05 ± 0.13	34.72 ± 0.47	< 0.001	22.72 ± 0.08	20.62 ± 0.42	< 0.001
MaxGS-nonDH (kg) ^***^	36.32 ± 0.13	32.67 ± 0.47	< 0.001	21.28 ± 0.08	19.61 ± 0.39	< 0.001
MaxGS-BHs (kg) ^***^	38.75 ± 0.13	35.17 ± 0.47	< 0.001	23.14 ± 0.08	21.12 ± 0.40	< 0.001
MeanGS-BHs (kg) ^***^	35.40 ± 0.13	31.93 ± 0.46	< 0.001	20.65 ± 0.08	18.73 ± 0.38	< 0.001
Relative HGS						
MaxGS-DH/BMI^***^	1.58 ± 0.01	1.43 ± 0.02	< 0.001	0.96 ± 0.00	0.82 ± 0.02	< 0.001
MaxGS-nonDH/BMI^***^	1.51 ± 0.01	1.35 ± 0.02	< 0.001	0.90 ± 0.00	0.78 ± 0.02	< 0.001
MaxGS-BHs/BMI^***^	1.61 ± 0.01	1.45 ± 0.02	< 0.001	0.98 ± 0.00	0.84 ± 0.02	< 0.001
MeanGS-BHs/BMI^***^	1.47 ± 0.01	1.32 ± 0.02	< 0.001	0.87 ± 0.00	0.74 ± 0.02	< 0.001
MaxGS-DH/WC^***^	0.44 ± 0.00	0.39 ± 0.01	< 0.001	0.28 ± 0.00	0.24 ± 0.01	< 0.001
MaxGS-nonDH/WC^***^	0.42 ± 0.00	0.37 ± 0.01	< 0.001	0.26 ± 0.00	0.23 ± 0.00	< 0.001
MaxGS-BHs/WC^***^	0.45 ± 0.00	0.40 ± 0.01	< 0.001	0.29 ± 0.00	0.25 ± 0.00	< 0.001
MeanGS-BHs/WC^***^	0.41 ± 0.00	0.36 ± 0.01	< 0.001	0.26 ± 0.00	0.22 ± 0.00	< 0.001
MaxGS-DH/WHtR^***^	74.23 ± 0.29	65.81 ± 1.00	< 0.001	43.75 ± 0.19	36.77 ± 0.83	< 0.001
MaxGS-nonDH/WHtR^***^	70.84 ± 0.29	62.02 ± 1.01	< 0.001	40.98 ± 0.19	34.91 ± 0.76	< 0.001
MaxGS-BHs/WHtR^***^	75.59 ± 0.29	66.69 ± 0.99	< 0.001	44.54 ± 0.19	37.65 ± 0.81	< 0.001
MeanGS-BHs/WHtR^***^	69.07 ± 0.28	60.58 ± 0.97	< 0.001	39.78 ± 0.19	33.36 ± 0.74	< 0.001
MaxGS-DH/Weight^***^	0.56 ± 0.00	0.51 ± 0.01	< 0.001	0.40 ± 0.00	0.35 ± 0.01	< 0.001
MaxGS-nonDH/Weight^***^	0.53 ± 0.00	0.48 ± 0.01	< 0.001	0.37 ± 0.00	0.33 ± 0.01	< 0.001
MaxGS-BHs/Weight^***^	0.57 ± 0.00	0.52 ± 0.01	< 0.001	0.40 ± 0.00	0.36 ± 0.01	< 0.001
MeanGS-BHs/Weight^***^	0.52 ± 0.00	0.47 ± 0.01	< 0.001	0.36 ± 0.00	0.32 ± 0.01	< 0.001

SBP: systolic blood pressure, DBP: diastolic blood pressure, HGS: hand grip strength, MaxGS-DH: maximum grip strength of the dominant hand, MaxGS-nonDH: maximum grip strength of the nondominant hand, MaxGS-BHs: maximum grip strength in both hands, MeanGS-BHs: mean grip strength of both hands, BMI: body mass index, WC: waist circumference, WHtR: waist-to-height ratio, MIAP: myocardial infarction/angina pectoris, IHD: ischemic heart disease

Continuous data are presented as the means ± SEs (standard errors). Categorical data are represented as percentages (SEs).

* *p* < 0.05, ** *p* < 0.01, *** *p* < 0.001. *, **, and *** indicate *p* values for sex differences between all men and women. *P* values were obtained from Rao–Scott chi-squared tests for categorical variables and from a general linear model for continuous variables between the MIAP group and the non-MIAP group

For anthropometric indices and blood pressure, significant differences in DBP (*p* < 0.001), height (*p* = 0.012), WC (*p* = 0.001), and the WHtR (*p* < 0.001) were found between the MIAP and non-MIAP groups for men, and all variables showed statistically significant between-group differences in women. Overall, the MIAP group had a lower DBP, shorter stature, greater WC, and greater WHtR than did the non-MIAP group for both men and women, and women tended to have higher SBP, weight, and BMI. Regarding HGS-related variables, significant differences were observed between the two groups in all HGS-related variables, excluding the dominant hand variable, for both men and women. The HGS of the MIAP group tended to be lower than that of the non-MIAP group, with greater differences observed in men than in women.

### Associations of MIAP with anthropometric indices and HGS

We created three models based on adjusted variables: the crude model was not adjusted; Model 1 was adjusted for age; and Model 2 was adjusted for age, residential area, education level, occupation type, household income, stress, alcohol consumption, smoking status, family history of IHD, resistance exercise, and walking exercise. Tables 2 and 3 show the associations of MIAP with anthropometric indices, absolute HGS indices, and relative HGS indices. In men, although most absolute and relative HGS indices were strongly associated with MAIP, relative HGS indices combined with WC and the WHtR had a greater association with MIAP than did anthropometric indices and absolute HGS indices in all crude analyses and in adjusted Models 1 and 2. MIAP showed a stronger negative association with MaxGS-nonDH/WC (adjusted odds ratio (adj. OR) = 0.68 [0.58–0.80], *p* = < 0.001), MaxGS-BHs/WC (adj. OR = 0.69 [0.58–0.81], *p* = < 0.001), MaxGS-nonDH/WHtR (adj. OR = 0.69 [0.58–0.82], *p* = < 0.001), and MaxGS-BHs/WHtR (adj. OR = 0.69 [0.58–0.83], *p* = < 0.001) compared to the other variables in Model 2. However, in women, anthropometric indices were more strongly associated with MIAP than were absolute and relative HGS indices. MIAP was more positively associated with weight (adj. OR = 1.41 [1.24–1.60], *p* = < 0.001), BMI (adj. OR = 1.44 [1.26–1.64], *p* = < 0.001), WC (adj. OR = 1.42 [1.23–1.63], *p* = < 0.001), and the WHtR (adj. OR = 1.42 [1.22–1.65], *p* = < 0.001) in adjusted Model 2. According to the comparisons between absolute and relative HGS indices, all absolute HGS indices and other relative HGS indices were strongly associated with MIAP, but all associations disappeared in adjusted models 1 and 2, except for 4 relative HGS combined with weight and BMI indices. Specifically, MIAP showed a negative association with MaxGS-DH/BMI (adj. OR = 0.82 [0.67-1.00], *p* = 0.047), MaxGS-DH/Weight (adj. OR = 0.81 [0.68–0.96], *p* = 0.017), MaxGS-BHs/Weight (adj. OR = 0.82 [0.69–0.97], *p* = 0.021), and MeanGS-BHs/Weight (adj. OR = 0.84 [0.71-1.00], *p* = 0.048) in adjusted Model 2. In both men and women, relative HGS was more strongly and more significantly associated with MIAP than was absolute HGS, even though anthropometric indices were more strongly associated with MIAP than were HGS indices in women.

**Table 2:** Associations of MIAP with anthropometric indices and absolute and relative HGS indices among men

Variables	Crude		Model 1		Model 2	
	OR (95% CI)	*p* value	OR (95% CI)	*p* value	OR (95% CI)	*p* value
Age	1.69 (1.49–1.93)	< 0.001		-		-
Anthropometrics						
Height	0.84 (0.73–0.96)	0.011	1.01 (0.87–1.17)	0.933	1.02 (0.88–1.18)	0.806
Weight	0.97 (0.85–1.12)	0.694	1.16 (1.02–1.33)	0.029	1.17 (1.02–1.34)	0.023
Body mass index	1.07 (0.95–1.21)	0.271	1.17 (1.04–1.31)	0.009	1.16 (1.04–1.31)	0.011
Waist circumference	1.25 (1.10–1.41)	0.001	1.24 (1.10–1.41)	0.001	1.22 (1.08–1.38)	0.002
Waist-to-height ratio	1.33 (1.18–1.50)	< 0.001	1.24 (1.10–1.40)	0.001	1.21 (1.08–1.37)	0.002
Absolute HGS						
MaxGS-DH	0.65 (0.57–0.73)	< 0.001	0.82 (0.70–0.95)	0.009	0.84 (0.72–0.98)	0.028
MaxGS-nonDH	0.61 (0.54–0.69)	< 0.001	0.75 (0.65–0.87)	< 0.001	0.76 (0.65–0.89)	0.001
MaxGS-BHs	0.62 (0.54–0.70)	< 0.001	0.77 (0.66–0.90)	0.001	0.78 (0.66–0.92)	0.004
MeanGS-BHs	0.62 (0.55–0.70)	< 0.001	0.77 (0.66–0.90)	0.001	0.79 (0.67–0.92)	0.004
Relative HGS						
MaxGS-DH/BMI	0.62 (0.55–0.70)	< 0.001	0.75 (0.65–0.87)	< 0.001	0.76 (0.66–0.88)	< 0.001
MaxGS-nonDH/BMI	0.59 (0.52–0.67)	< 0.001	0.70 (0.61–0.81)	< 0.001	0.71 (0.61–0.83)	< 0.001
MaxGS-BHs/BMI	0.59 (0.52–0.67)	< 0.001	0.71 (0.61–0.82)	< 0.001	0.72 (0.61–0.83)	< 0.001
MeanGS-BHs/BMI	0.60 (0.53–0.68)	< 0.001	0.72 (0.62–0.83)	< 0.001	0.73 (0.62–0.85)	< 0.001
MaxGS-DH/WC	0.59 (0.53–0.67)	< 0.001	0.72 (0.62–0.84)	< 0.001	0.74 (0.63–0.86)	< 0.001
MaxGS-nonDH/WC	0.57 (0.50–0.64)	< 0.001	0.68 (0.58–0.79)	< 0.001	0.68 (0.58–0.80)	< 0.001
MaxGS-BHs/WC	0.57 (0.50–0.64)	< 0.001	0.68 (0.58–0.79)	< 0.001	0.69 (0.58–0.81)	< 0.001
MeanGS-BHs/WC	0.58 (0.51–0.65)	< 0.001	0.69 (0.59–0.80)	< 0.001	0.70 (0.59–0.82)	< 0.001
MaxGS-DH/WHtR	0.59 (0.52–0.67)	< 0.001	0.73 (0.62–0.85)	< 0.001	0.74 (0.63–0.88)	< 0.001
MaxGS-nonDH/WHtR	0.57 (0.50–0.65)	< 0.001	0.68 (0.58–0.80)	< 0.001	0.69 (0.58–0.82)	< 0.001
MaxGS-BHs/WHtR	0.57 (0.50–0.65)	< 0.001	0.68 (0.58–0.81)	< 0.001	0.69 (0.58–0.83)	< 0.001
MeanGS-BHs/WHtR	0.58 (0.50–0.65)	< 0.001	0.70 (0.59–0.82)	< 0.001	0.71 (0.59–0.84)	< 0.001
MaxGS-DH/Weight	0.64 (0.57–0.72)	< 0.001	0.76 (0.66–0.86)	< 0.001	0.76 (0.67–0.87)	< 0.001
MaxGS-nonDH/Weight	0.61 (0.54–0.69)	< 0.001	0.71 (0.62–0.81)	< 0.001	0.71 (0.62–0.82)	< 0.001
MaxGS-BHs/Weight	0.61 (0.54–0.69)	< 0.001	0.72 (0.62–0.82)	< 0.001	0.72 (0.62–0.83)	< 0.001
MeanGS-BHs/Weight	0.61 (0.55–0.69)	< 0.001	0.72 (0.63–0.83)	< 0.001	0.73 (0.63–0.84)	< 0.001

HGS: hand grip strength, MaxGS-DH: maximum grip strength of the dominant hand, MaxGS-nonDH: maximum grip strength of the nondominant hand, MaxGS-BHs: maximum grip strength of both hands, MeanGS-BHs: mean grip strength of both hands, BMI: body mass index, WC: waist circumference, WHtR: waist-to-height ratio, MIAP: myocardial infarction/angina pectoris, IHD: ischemic heart disease, OR: odds ratio, CI: confidence interval

ORs and *p* values were obtained from the crude and adjusted analyses using complex sample binary logistic regression. Odds ratios were estimated with 95% confidence intervals

Model 1 was adjusted for age

Model 2 was adjusted for age, residential area, education level, occupation type, household income, stress, alcohol consumption, smoking status, family history of IHD, resistance exercise, and walking exercise

**Table 3:** Associations of MIAP with anthropometric indices and absolute and relative HGS indices among women

Variables	Crude		Model 1		Model 2	
	OR (95% CI)	*p* value	OR (95% CI)	*p* value	OR (95% CI)	*p* value
Age	2.41 (2.09–2.79)	< 0.001	-	-	-	-
Anthropometrics						
Height	0.69 (0.60–0.79)	< 0.001	1.06 (0.89–1.26)	0.522	1.07 (0.90–1.28)	0.451
Weight	1.19 (1.05–1.34)	0.005	1.40 (1.24–1.59)	< 0.001	1.41 (1.24–1.60)	< 0.001
Body mass index	1.47 (1.31–1.64)	< 0.001	1.42 (1.25–1.61)	< 0.001	1.44 (1.26–1.64)	< 0.001
Waist circumference	1.65 (1.46–1.87)	< 0.001	1.41 (1.23–1.62)	< 0.001	1.42 (1.23–1.63)	< 0.001
Waist-to-height ratio	1.81 (1.60–2.04)	< 0.001	1.40 (1.21–1.62)	< 0.001	1.42 (1.22–1.65)	< 0.001
Absolute HGS						
MaxGS-DH	0.67 (0.57–0.78)	< 0.001	1.05 (0.87–1.28)	0.617	1.05 (0.86–1.27)	0.647
MaxGS-nonDH	0.71 (0.61–0.83)	< 0.001	1.13 (0.93–1.38)	0.225	1.14 (0.93–1.40)	0.209
MaxGS-BHs	0.67 (0.58–0.78)	< 0.001	1.07 (0.88–1.31)	0.487	1.07 (0.88–1.31)	0.488
MeanGS-BHs	0.68 (0.58–0.79)	< 0.001	1.09 (0.89–1.32)	0.406	1.09 (0.89–1.33)	0.417
Relative HGS						
MaxGS-DH/BMI	0.56 (0.48–0.65)	< 0.001	0.83 (0.68-1.00)	0.052	0.82 (0.67-1.00)	0.047
MaxGS-nonDH/BMI	0.59 (0.51–0.69)	< 0.001	0.88 (0.73–1.06)	0.183	0.88 (0.72–1.07)	0.183
MaxGS-BHs/BMI	0.55 (0.47–0.65)	< 0.001	0.83 (0.68–1.01)	0.059	0.82 (0.67–1.01)	0.056
MeanGS-BHs/BMI	0.57 (0.49–0.66)	< 0.001	0.85 (0.70–1.03)	0.102	0.84 (0.69–1.03)	0.093
MaxGS-DH/WC	0.55 (0.47–0.64)	< 0.001	0.86 (0.71–1.04)	0.126	0.85 (0.69–1.04)	0.119
MaxGS-nonDH/WC	0.58 (0.51–0.68)	< 0.001	0.92 (0.76–1.11)	0.378	0.92 (0.75–1.12)	0.382
MaxGS-BHs/WC	0.55 (0.47–0.64)	< 0.001	0.86 (0.71–1.05)	0.148	0.86 (0.70–1.06)	0.147
MeanGS-BHs/WC	0.56 (0.49–0.65)	< 0.001	0.89 (0.73–1.08)	0.223	0.88 (0.72–1.07)	0.208
MaxGS-DH/WHtR	0.54 (0.46–0.63)	< 0.001	0.86 (0.70–1.06)	0.160	0.86 (0.69–1.06)	0.159
MaxGS-nonDH/WHtR	0.57 (0.49–0.66)	< 0.001	0.92 (0.75–1.13)	0.414	0.92 (0.74–1.14)	0.432
MaxGS-BHs/WHtR	0.53 (0.46–0.62)	< 0.001	0.87 (0.70–1.07)	0.185	0.86 (0.69–1.08)	0.191
MeanGS-BHs/WHtR	0.54 (0.47–0.63)	< 0.001	0.89 (0.72–1.09)	0.251	0.88 (0.71–1.09)	0.245
MaxGS-DH/Weight	0.60 (0.52–0.70)	< 0.001	0.82 (0.69–0.97)	0.021	0.81 (0.68–0.96)	0.017
MaxGS-nonDH/Weight	0.64 (0.55–0.74)	< 0.001	0.88 (0.74–1.03)	0.113	0.87 (0.74–1.03)	0.103
MaxGS-BHs/Weight	0.60 (0.52–0.70)	< 0.001	0.82 (0.70–0.98)	0.025	0.82 (0.69–0.97)	0.021
MeanGS-BHs/Weight	0.61 (0.53–0.71)	< 0.001	0.85 (0.72–1.01)	0.059	0.84 (0.71-1.00)	0.048

HGS: hand grip strength, MaxGS-DH: maximum grip strength of the dominant hand, MaxGS-nonDH: maximum grip strength of the nondominant hand, MaxGS-BHs: maximum grip strength of both hands, MeanGS-BHs: mean grip strength of both hands, BMI: body mass index, WC: waist circumference, WHtR: waist-to-height ratio, MIAP: myocardial infarction/angina pectoris, IHD: ischemic heart disease, OR: odds ratio, CI: confidence interval

ORs and *p* values were obtained from the crude and adjusted analyses using complex sample binary logistic regression. Odds ratios were estimated with 95% confidence intervals

Model 1 was adjusted for age

Model 2 was adjusted for age, residential area, education level, occupation type, household income, stress, alcohol consumption, smoking status, family history of IHD, resistance exercise, and walking exercise

## Discussion

In this large-scale cross-sectional study, we examined the associations of MIAP with anthropometric indices, absolute HGS indices, and relative HGS indices. We found that HGS was strongly associated with the risk of MIAP in the Korean population. MIAP might be better identified by relative HGS than absolute HGS in both sexes, but anthropometric indices were more strongly associated with MIAP than HGS indices in women. The overall magnitudes of the associations of MIAP with absolute and relative HGS are greater in men than in women.

Generally, the risk factors for MIAP include increasing age [45]; sex and ethnicity [46]; a history of cardiovascular diseases (CVDs) [45–47]; hypertension [9, 45–47]; diabetes [9, 45–47]; dyslipidemia [46]; abnormal serum lipid levels such as proinsulin, C-reactive protein (CRP), uric acid, high-density lipoprotein cholesterol (HDL) cholesterol, and non-HDL cholesterol levels [9, 32, 47–50]; genetics [46]; smoking [9, 46–48, 51]; obesity [9, 46, 47, 51]; alcohol consumption [9, 47, 51, 52]; high DBP [48]; low physical activity or heavy exercise [9, 46, 47]; oxidative stress [51]; low education level [9]; psychosocial factors [47]; and low HGS [4–7, 9, 10]. However, these risk factors may differ according to sex, ethnic group or country. For example, low alcohol consumption is related to a low or moderate reduction in MI risk, but the protective effect of low alcohol consumption is greater in women than in men [52]. Additionally, a protective effect has been observed in many countries but not in South Asian countries such as India, Pakistan, Nepal, or Bangladesh [52]. Furthermore, the mean age of onset of MI was approximately 9 years later in men than in women in many ethnic groups and countries [47].

To date, an association between HGS and MIAP has been reported in various ethnic groups and countries. Leong et al. [4] examined the association of HGS with MI risk, stroke risk, and cardiovascular mortality risk in 139,691 subjects across 17 high-income and low-income countries. They reported that HGS was inversely associated with MI and stroke risk after adjustment for various potential confounders and argued that HGS was a predictor of death in patients with cardiovascular or noncardiovascular disease. Chainani et al. [6] assessed the association of cardiovascular mortality with HGS and gait speed via a systematic review and argued that low HGS and gait speed were linked to a high risk of cardiovascular mortality across diverse populations. Additionally, Lopez-Jaramillo et al. [7] investigated the relationship between HGS and CVDs such as MI, stroke, or death in patients with prediabetes and diabetes and documented that higher HGS was related to a lower incidence of death and cardiovascular events in both men and women, irrespective of adiposity and whether they resided in a high- or low-income country. Park et al. [8] examined the causal effect of HGS and walking pace on MI and CVD risk based on observational investigations and genetic instruments and reported that observational and genetically predicted low HGS and slow walking pace predicted the risk of MI or cardiovascular mortality. Yusuf et al. [9] tested the association of HGS with MI, stroke, and CVD risk based on data from the Prospective Urban Rural Epidemiology (PURE) study and reported that low HGS was a risk factor for MI, CVD, and stroke. Xu and Hao examined the causal effect of HGS on MI and CAD risk using single nucleotide polymorphisms (rs3121278 and rs752045) as genetic instruments for HGS in a Mendelian randomization study [10]. They argued that an increase of 1 kg in genetically determined HGS decreased the odds of MI by 7%. In the Korean population, several studies have examined the association of CVDs with absolute and relative HGS [11, 12]. Jang et al. [12] evaluated the association of heart disease (MIAP and congestive heart failure) with absolute and relative HGS through a longitudinal study. After adjustment for various confounders, they argued that relative HGS (HGS/BMI) was more strongly associated with heart disease than were absolute and dominant HGS in both men and women. Kim et al. [11] examined the association between relative HGS and cardiometabolic risk factors such as MIAP, hypertension, type 2 diabetes, osteoporosis, and obesity. They documented that MIAP was associated with relative HGS in a crude analysis but not in an adjusted analysis. These studies only used HGS/BMI as a relative HGS index [11, 12]; therefore, the results of these studies were limited by comparing only absolute HGS and one relative HGS index (HGS/BMI). A comparison of our findings with the results of previous studies showed that our findings were consistent with the results of previous studies [4, 6, 7, 9, 11, 12], indicating that low HGS was significantly related to the risk of MIAP or CVDs. Additionally, we agreed that relative HGS was superior to absolute or dominant HGS, as shown in the results of a previous study [12]. However, our findings differed from the results of other previous studies [11]. The results of our study showed that relative HGS indices combined with BMI and weight were significantly associated with MIAP in both men and women according to both the crude and adjusted models. We assume that the reasons for this discrepancy are the differences in the use of relative HGS indices, target diseases, or adjustment for covariates.

More recently, several studies have emphasized the usefulness of relative HGS indices combined with BMI, WC, weight, height, and fat mass in the identification of several diseases [13–15, 20]. Kis et al. [13] evaluated the predictive power of relative HGS indices for identifying older patients with type 2 diabetes and reported that the HGS/WC index was the best predictor of type 2 diabetes in older patients. Lee et al. [14] examined the association of cardiometabolic risk factors with relative HGS in Taiwanese men and women and argued that the HGS/BMI was a better indicator of cardiometabolic health than was absolute HGS. Similarly, Lawman et al. [15] tested the association between HGS and cardiovascular health biomarkers in U.S. adults and documented that the HGS/BMI was more strongly associated with cardiovascular biomarkers than was absolute HGS. Byeon et al. [20] examined the relationship of metabolic syndrome with absolute HGS and relative HGS in Korean adults and demonstrated that the HGS/weight index was more strongly associated with the incidence of metabolic syndrome than was absolute HGS. Although several studies have reported an association between the relative HGS index and the risk of several diseases, no studies have reported an association between relative HGS and MIAP.

The pathological mechanism by which low HGS is associated with an increased risk of MIAP, CVD, and mortality is unclear [4, 12], even though HGS is useful as a simple and inexpensive indicator of many diseases. However, possible explanations or mechanisms for the association can be suggested. First, our results revealed sex-specific differences in that the association between MIAP and HGS persisted in all the adjusted models for men, but for women, the association disappeared in the age-adjusted model. There are sex-specific differences in HGS according to age, hormonal changes, blood profile, and disease, such as arthritis and stroke [11, 44, 53]. A reduction in HGS was associated with aging and hormonal imbalances in women [44, 53]. Additionally, the sex-specific factors associated with HGS were diabetes, stroke, or osteoporosis in men and osteoarthritis in women [54]. HGS was associated with triglyceride and high-density lipoprotein levels in men and fasting plasma glucose levels in women [55]. In Korea, the prevalence of arthritis was much greater in women than in men [44]. In contrast, MIAP was more prevalent in men than in women [11]. Additionally, muscle strength and hormonal changes during aging differ according to sex [11]. The frequency of muscular and resistance exercise was much greater in men than in women in this study. We assumed that these conditions may induce sex differences in the association between HGS and MIAP. However, the cause of these sex differences is still unclear, and further studies are needed. Second, relative HGS was negatively associated with insulin resistance in both sexes [11]. Insulin resistance was linked to independent cardiovascular risk factors, including MIAP, due to its association with inflammation, such as glycometabolic abnormalities, high-sensitivity C-reactive protein (hs-CRP) levels, and fibrinogen levels [50, 56, 57]. For example, Hs-CRP, an inflammatory index, has a negative effect on relative HGS [11]. The biological mechanism underlying the relationship between chronic inflammation and low physical function was explained by the fact that inflammatory markers such as high CRP and interleukin-6 levels are inversely and independently related to poor physical performance and HGS [57]. Inflammation is the body’s defense response against disorders of homeostasis due to a local release of cytokines [57]. Cytokines are related to physiological functions such as muscle tissue turnover and immunoregulation, and their circulating levels are associated with CVD [57, 58]. Additionally, obesity and visceral adiposity are causally associated with insulin resistance [11, 59] and were suggested to be risk factors for MIAP in previous studies [9, 46, 47, 51]. Like these studies, the present study demonstrated that obesity indices such as weight, BMI, WC, and the WHtR were strongly associated with MIAP in both men and women according to the adjusted models. Sarcopenia and sarcopenic obesity are associated with a high risk of cardiometabolic and musculoskeletal diseases and reduced muscle mass and strength [11, 60, 61]. Furthermore, HGS is closely related to DBP, total cholesterol, LDL-cholesterol, and triglyceride levels [11, 61], and these markers are known to be risk factors for MIAP [9, 32, 45–50].

Our study has several limitations. We cannot establish a cause‒effect relationship due to the cross-sectional design. Additionally, data on the diagnosis of MIAP were obtained via questionnaires. Therefore, to overcome respondent recall bias in the diagnosis of MIAP, a health interview survey was performed through a face-to-face interview with experts and well-trained staff according to specific guidelines [29–31]. Despite these limitations, the statistical results of this study are strong and powerful because the very large KNHANES dataset includes a nationally representative sample of the Korean population. To our knowledge, this is the first study to compare anthropometric indices, absolute HGS indices, and relative HGS indices and their associations with MIAP risk in a large population-based investigation.

In conclusion, we examined the associations of MIAP with anthropometric indices, absolute HGS indices, and relative HGS indices. In men, the relative HGS indices combined with WC and the WHtR had greater associations with MIAP than did the anthropometric and absolute HGS indices. However, anthropometric indices were more strongly associated with MIAP than were absolute and relative HGS indices in women. When comparing absolute and relative HGS indices in women, except for anthropometric indices, relative HGS indices combined with BMI and body weight were more strongly related to MIAP than were absolute HGS indices.

## Data Availability

Data used in this study are available from the Korea National Health and Nutrition Examination Survey (KNHANES) performed by the Korea Centers for Disease Control and Prevention (KCDC). Anyone can freely access the data (https://knhanes.kdca.go.kr/knhanes/sub03/sub03_02_05.do).

## Abbreviations

HGS: hand grip strength
MaxGS-DH: maximum grip strength of the dominant hand
MaxGS-nonDH: maximum grip strength of the nondominant hand
MaxGS-BHs: maximum grip strength of both hands
MeanGS-BHs: mean grip strength of both hands
BMI: body mass index
WC: waist circumference
WHtR: waist-to-height ratio
MIAP: myocardial infarction/angina pectoris
MI: myocardial infarction
IHD: ischemic heart disease
SBP: systolic blood pressure
DBP: diastolic blood pressure
ACS: acute coronary syndrome
